# Medical Diagnoses, Mode of Residence, and Dental Treatment Demand under General Anesthesia in Special Needs Adults in Innsbruck, Austria. A Retrospective Breakdown of Four and a Half Years

**DOI:** 10.3390/healthcare9030279

**Published:** 2021-03-04

**Authors:** Dagmar Schnabl, Michael Oberhofer, Fabian Barbieri, Johannes Laimer, René Steiner, Emanuel Bruckmoser, Ingrid Grunert

**Affiliations:** 1 Department of Operative and Prosthetic Dentistry, Medical University Innsbruck, 6020 Innsbruck, Austria; michael.oberhofer@student.i-med.ac.at (M.O.); johannes.laimer@i-med.ac.at (J.L.); rene.steiner@tirol-kliniken.at (R.S.); ingrid.grunert@i-med.ac.at (I.G.); 2 Department of Internal Medicine III, Cardiology and Angiology, Medical University Innsbruck, 6020 Innsbruck, Austria; fabian.barbieri@tirol-kliniken.at; 3 Private Practice for Oral and Maxillofacial Surgery, 5020 Salzburg, Austria; research@bruckmoser.info

**Keywords:** adults, dental general anesthesia, special needs, intellectual disablement, physical disablement, psychiatric disorder, dentist phobia, nursing home, home health care

## Abstract

Regarding oral/dental care and attendance, special needs individuals depend on their caregivers’ commitment. The purpose of this retrospective data analysis of adults who received dental general anesthesia (DGA) in Innsbruck, Austria, was a breakdown of demographic parameters (including the mode of accommodation/care), medical diagnoses (comprising intellectual/physical disablement (IPD) or psychiatric (anxiety) disorders (PDs)), and dental therapy performed under DGA. The sample was composed of 233 consecutive adults who underwent DGA from January 2015 to June 2019. Data were analyzed with descriptive and comparative statistics. In total, 133 (57.1%) subjects were male and 100 (42.9%) female; 176 (75.5%) had IPD and 57 (24.5%) PDs; 168 (72.1%) were living at private and 65 (27.9%) at nursing homes. Median age (IQR) was 35.6 (25.7–47.2) years. In the total sample, 5 (2–9) teeth were restored and 2 (0.5–6.5) teeth were extracted. Individuals with PDs had more teeth restored (*p* = 0.01) and extracted (*p* < 0.001) than individuals with IPD. Private home residents had more teeth restored (*p* < 0.001) but less teeth extracted (*p* = 0.003) than nursing home residents. Special needs individuals’ oral health backlog should be tackled in private and institutional care modalities alike.

## 1. Introduction

“Disablement pertinent to dentistry” has been defined as a physical or mental condition that impedes the ability to perform oral hygiene or to cooperate with dental treatment [1]. As a consequence of insufficient dental care, oral health outcomes (including periodontal disease, caries, and tooth loss) are poor in individuals with mental and developmental disabilities [2,3,4]. Regarding the performance of domestic oral hygiene and the keeping of dentist appointments, individuals with special needs to a greater or lesser extent depend on their caregivers’ commitment. According to the nature (physical, intellectual, psychological, or combined) and the severity of the respective disablement, daily teeth cleaning needs to be either assisted or carried out completely by attending private carers or professional nursing staff. While in private caretaking, usually a small number of carers or family members are in charge of one individual, changing staff share attendance of multiple foster home residents within a shift system. Therefore, emotional attachment and approach to care recipients are likely to somewhat differ between private carers and professionals. In any case, oral hygiene should be based on professional expertise and the needs and preferences of the individual with disabilities and his/her carers [5]. Professional domiciliary oral hygiene services (which are, to the authors’ knowledge, largely lacking in Austria) might thereby increase patient acceptance for routine dental care [6,7,8].

Aside from a hampered domestic oral hygiene, limited access to dental treatment has been identified as a cause for a backlog in oral health in children and adults with special needs [9,10,11]. Physical inaccessibility to oral care facilities, lack of access to information among carers, lack of knowledge of disability issues, and inadequate experience and skills in special needs dentistry among dental practitioners are the key barriers to dental service use [10,12].

Due to anxiety, non-cooperativeness, or defense, dental treatment of severely impaired individuals with special needs poses a challenge and is frequently not contrivable under office conditions. In these cases, dental general anesthesia (DGA) is a commonly used approach to provide optimized treatment conditions [13,14]. Under defined precautions (including a thorough pre-operative medical assessment), DGA has been considered a safe procedure when carried out by a specialized and well-equipped team [13,14,15,16], as is the case at the University Hospital of Innsbruck, Austria. However, particularly in medically compromised individuals, a certain risk of peri- or postoperative anesthetic complications remains [13,17]. Another issue to be regarded with the accomplishment of DGA is the expense factor. Costs for DGA by far exceed those of preventive measures or conventional dental therapy [18,19]. In light of these drawbacks, the provision of DGA should be kept to imperative necessity.

The purpose of this retrospective analysis of demographic parameters (including the mode of accommodation and care, medical diagnoses, and treatment performed under general anesthesia (GA)) in adults with special needs was to contribute relevant data to oral health policies. We aimed to provide a breakdown of diagnoses necessitating DGA in adults and to assess the impact of residence (private versus institutional) on the dental treatment demand. Our results shall serve as a basis for the discussion of whether certain preventive and therapeutic measures could reduce the demand for DGA in this special clientele.

## 2. Material and Methods

### 2.1. Subjects

The study sample was composed of all consecutive patients older than 16 years of age who underwent scheduled DGA at the University Clinic of Dental Prosthetics and Restorative Dentistry of Innsbruck from 1 January 2015 to 30 June 2019. The cohort consisted of patients displaying decayed and/or periodontally compromised teeth in need of therapy, who were unamenable to a conventional treatment approach under local anesthesia due to substantial intellectual or physical disablement (IPD) or psychiatric disorders (PDs) with inherent (dental) anxiety. Patients were either referred to DGA by their dentists (whose expertise and equipment with regard to dealing with special needs patients are unknown but supposedly limited) after unsuccessful treatment attempts or, in cases of evident non-cooperativeness or defense, by their physicians. Some patients presented on their own or their caregivers’ initiative. Obvious carious decay, broken or loose teeth, or presumed pain were frequent reasons for seeking consultation. Patients with PDs had to submit a psychiatrist’s note that confirmed the impracticability of dental treatment under office conditions. At admission, according to the respective patient’s cooperativeness, an enoral clinical examination was performed by the consultant in charge and an orthopantomogram was taken if possible and not provided by the referring dentist. A preliminary treatment plan was made as far as contrivable. Informed consent to DGA was obtained from legally competent patients or patients’ statutory representatives.

### 2.2. Dental Treatment under General Anesthesia

Prior to the scheduled intervention, contraindications to GA were ruled out through an internistic checkup at the outpatient clinic of the Department of Anaesthesiology and Critical Care Medicine. DGA was accomplished in the course of a day-unit stay (including postoperative care at a postanesthesia care unit). Under endotracheal anesthesia, which is a recommended method to prevent complications associated with airway management [13], teeth were professionally cleaned and a thorough clinical assessment with respect to carious or periodontally compromised teeth was made. However, neither the decayed-missing-filled teeth (DMFT) index nor probing depths/bleeding on probing were recorded. If no preoperative orthopantomogram was available, intraoral radiographs were taken. The original treatment plan was revised accordingly. Carious teeth were restored by means of composite. Loose and profoundly carious teeth presenting vital or avital pulp exposition on excavation were extracted. Although single-visit endodontic therapy under GA has been proven feasible [20], this treatment option has been refrained from at the University Hospital of Dental Prosthetics and Restorative Dentistry of Innsbruck in order to keep the duration of GA short and to ensure postoperative long-term absence of pain. According to internal directives, the risk of endodontic complications [21] has been avoided so as to prevent painful conditions necessitating further DGA. This approach is backed up by the immanent risk of anesthetic complications of DGA in special needs patients [13,17] and the hospital’s limited capacity for (repeated) DGA. The treatment goal was the best possible elimination of dental pathologies. After discharge, patients were either retransferred to their referring dentists or invited to make use of a program dedicated to special needs patients at the University Hospital of Dental Prosthetics and Restorative Dentistry. In the course of this program, half-year recall intervals are envisaged. According to cooperation, every Tuesday, scheduled professional oral hygiene, restorative dental treatment, and prosthetic rehabilitation (mostly removable) are provided by advanced dental students.

### 2.3. Study Design and Data Acquisition

The present study was conducted in the year 2019 in accordance with the guidelines of the local ethics board and the Helsinki Declaration of 1975, as revised in 2013. In Austria, de jure, ethical approval and informed consent are not required for retrospective analysis (and publication) of anonymized data retrieved from patient files. This has been confirmed by a written general statement issued to the authors by the Ethics Committee of the Medical University of Innsbruck. Pseudonymized data collection from electronical patient records and data analysis were performed in the course of a thesis that was approved by the Medical University of Innsbruck.

The following data were gathered: age at the time of DGA, gender, medical or psychiatric diagnosis, type of residence (private home or nursing home), mode of referral, number of teeth and surfaces restored, and number of teeth extracted under DGA. In cases of multimorbidity, the severest diagnosis was listed.

### 2.4. Data Analysis

Standard descriptive statistics were used to summarize the data. Qualitative variables are reported as absolute and relative frequencies and quantitative variables as median (interquartile range (IQR)). The distribution of continuous variables was determined by the Kolmogorov–Smirnov test. In cases of a normal data distribution, the *t*-test, and in cases of not normally distributed data, the Mann–Whitney *U* test were used to assess statistical significance in differences. Comparisons of qualitative variables were calculated by using the Chi-square test. Statistical analysis was conducted by use of the IBM SPSS version 21 (IBM Corporation, Armonk, NY, USA). *p*-values ≤ 0.05 were regarded as statistically significant.

## 3. Results

### 3.1. Subjects

During the represented period of four and a half years, 233 adults underwent scheduled DGA. In total, 133 subjects (57.1%) were male and 100 (42.9%) were female.

A total of 176 individuals (75.5%) had an IPD and 57 (24.5%) were diagnosed with a PD. Table 1 and Table 2 list the detailed diagnoses that called for GA to facilitate dental treatment in subjects with IPD and individuals with PDs, respectively.

A total of 168 persons (72.1%) were living at private homes and 65 (27.9%) were accommodated at nursing homes.

Median age (IQR) at the time of DGA was 35.6 (25.7–47.2) years in the total collective.

Differences in age were significant between subjects with IPD and subjects with PDs (37.1 (26.8–51.2) versus 31.7 (25.5–38.2) years; *p* = 0.023) and between individuals living at private and individuals living at nursing homes (31.9 (23.8–39.5) versus 48.5 (34.2–59.2) years; *p* < 0.001).

While differences in gender were not significant between privately accommodated individuals and nursing home residents (*p* = 0.976), differences in diagnoses were highly significant between the two residence groups. In total, 65.3% of the IPD group and 93% of the PD group were accommodated at private homes (*p* < 0.001).

Ninety-six patients (41.2%) were referred to DGA by general practitioners and 83 (35.6%) by dentists. Eighty-three patients (35.6%) were referred by physicians of other medical specialties or presented on their own or their carers’ accord. Patients with PDs (n = 57) had to furnish a psychiatrist’s certificate affirming the necessity of GA for dental therapy.

### 3.2. Treatment under DGA

In the total sample, a median (IQR) of 5 (2–9) teeth and 9 (4–15) tooth surfaces were treated with composite restorations and 2 (0.5–6.5) teeth were extracted.

Table 3 summarizes dental therapy under GA by gender, diagnoses (IPD versus PD), and residence (private versus nursing home).

## 4. Discussion

Dental treatment demand (restoration of 5 (2–9) carious teeth and extraction of 2 (0.5–6.5) teeth with deep decay or severe periodontal damage) was high in our study sample as compared to the general population. An Austrian survey conducted in the year 2000 found a mean DMFT score of 14.7 (DT 0.2; MT 2.2; FT 12.3) in 35- to 44-year-olds [22]. According to the Fifth German Oral Health Study (2014), the DMFT Index was 11.2 in adults aged 35 to 44 years. Half a carious tooth per individual was in need of therapy [23]. Another German study compared clinical and radiological findings in special needs patients (collected in the years 1995 to 2003) with data of the average population (assessed in 1997) [2,24]. In the age group of 35- to 44-year-olds, the DMFT score was 16.2 (DT 4.3; MT 6.9; FT 5) in the special needs sample and 16.1 (DT 0.5; MT 3.9; FT 11.7) in the sample of the general population. This comparison outlines the difference in treatment demand between the two groups at a similar total DMFT score. Dental anxiety, a lack of insight into the importance of dental attendance, or an impaired mobility were discussed as reasons why special needs persons avail dental care more scarcely and, in many cases, only in the presence of discomfort or pain [2]. A Belgian study compared data on oral health-care utilization of adults with disabilities and adults without special needs. They recorded significantly fewer radiographs, restorations, and endodontic treatment and significantly more emergency visits in the special needs group and concluded that further research is needed to evaluate whether these findings point to high unmet oral-treatment needs [9].

The present investigation of scheduled treatment under DGA assessed significantly higher restoration and extraction rates in individuals with PDs compared to subjects with IPD at a significantly younger age of PD group. Procrastination of dental consultations due to dental phobia and perhaps the predominantly psychological support of persons with PDs (as opposed to a comprehensive nursing care in individuals with IPD) seem to result in the most disastrous oral health conditions. A recent Swiss study, for comparison, assigned adults, who were treated under DGA, to four groups (disability, dementia, dental phobia, and addictions/psychosocial disorders) and (conformingly to our study) found an overall high treatment need. Persons with dementia had the highest demand for restorations and persons with addiction/psychosocial disorders had the most decayed teeth [25]. A breakdown of treatment measures under DGA in special needs patients at the Helsinki Public Dental Service revealed that (in agreement with the present study) restorations constituted the highest share (57%), followed by tooth extractions (24%), preventive measures (5%), radiography (5%), endodontics (4%), and periodontics, surgical procedures, and other interventions (5%) [26].

With regard to residence, our analysis yielded differentiated results. Inhabitants of nursing homes had significantly more teeth extracted than individuals that were cared for privately (4 (1–9.5) versus 2 (0–5)). On the other hand, the prevalence of carious lesions in need of restorative therapy was significantly higher in residents at private homes as compared to those at nursing homes (6 (3–10) versus 3 (1–6) restored teeth and 11 (6–16) versus 7 (2–11) restored tooth surfaces). A possible explanation for the apparent discrepancy in the demand for extractions and restorations between the two groups might be the significantly higher age of the nursing home group as compared to the private home group (48.5 (34.2–59.2) versus 31.9 (23.8–39.5) years). It also seems that private carers tend to initiate dental attendance a little earlier (that is not only in the presence of obvious deep decay) than professional nursing home staff. Some previous studies have levelled criticism against the practice of oral health care at public nursing homes. Severe shortcomings of tooth and denture hygiene and the state of restorative and/or prosthodontic rehabilitation in geriatric samples at a mean age of >80 years were reviewed [27,28,29]. Staff shortage, an extensive workload, and insufficient training in oral care might account for these deficiencies at public foster homes. In private home care, family members’ exhaustion and overload as a corollary of 24/7 informal caregiving might contribute to a high prevalence of carious lesions. Special needs individuals’ ability to cooperate with oral interventions certainly depends on the nature and severity of the respective disablement or disease. The vast majority of our IPD group suffered from neurological disorders associated with intellectual deficits entailing incomprehension and anxiety (Table 1). Only few individuals were afflicted with mere bodily defects that affected cooperativeness with dental hygiene and treatment.

Domestic oral hygiene and the frequency of dental consultations should be personalized in special needs patients, based on specialized professional expertise according to individual requirements. For this purpose, professional and private carers should be trained alike. The implementation of professional domiciliary oral hygiene (in both private and institutional homes) might improve patients’ acceptance for dental home care and oral health outcomes [6,7,8]. On this understanding, the demand for DGA, which implicates a certain risk of peri- or postoperative complications as well as a high financial expenditure [13,17,18,19], might be reduced in patients with (moderately severe) IPD. In individuals with the severest IPD that are completely unamenable to any oral intervention, DGA constitutes the most feasible method for diagnostic, preventive, and therapeutic measures. In the PD group, treatment of the underlying psychiatric or psychological disorder and a considerate approach perhaps by use of alternative methods (such as behavior management, hypnodontia, or conscious sedation) might facilitate dental treatment under local anesthesia instead of or following DGA. To this effect, dentists’ training in special needs dentistry should be promoted.

According to a questionnaire survey, Australian care recipients in institutions and community housing had better access to dental care than those at family homes [12]. In Tirol, Austria, in any region, access to dental care providers is achievable within a reasonable distance and a referral to dental consultation or admission to DGA at the University Hospital of Innsbruck seems to be mostly a question of logistic effort. A well-working patient-transfer ambulance system is (at the instance of family or institutional physicians) available to private carers and public nursing homes.

The retrospective design of this study accounts for some limitations: data on patients’ oral hygiene and periodontal status, DMFT index, previous dental treatment, the severity of disablement, the modalities of oral care, the frequency of dental consultations, and the mode of aftercare were not (consistently) available from patient records. Another limitation might be the rather small study sample. The authors are aware that the classification of the registered diagnoses is—due to partial unspecificity—somewhat vague. However, our list of diagnoses elucidates the spectrum of conditions that impede oral care and call for dental treatment under DGA.

## 5. Conclusions

The present study revealed a high dental treatment demand in our clientele of special needs patients. Within our sample, individuals with PDs required more treatment than those suffering from IPD, and private home residents required more restorative therapy but less tooth extractions than nursing home residents.

Special needs individuals’ oral health backlog should be approached by promoting private and institutional carers’ training in domestic oral health care and by improving dental attendance.

In cases of severe impairment and non-cooperation with oral interventions, DGA is a feasible treatment method.

Further studies are needed to gather data on the modalities of oral care with respect to private or institutional residence and the respective oral health outcomes.

## Figures and Tables

**Table 1:** Diagnoses in subjects with intellectual or physical disablement (*n* = 176).

Number (Percentage)	Diagnosis
63 (35.8%)	Seizure disorders associated with intellectual (and possibly physical) disablement
55 (31.3%)	Intellectual (and possibly physical) disablement not further specified
24 (13.6%)	Chromosomal disorders and hereditary diseases with inherent developmental physical and/or mental disturbances
12 (6.8%)	Dementia
7 (4%)	Apallic syndrome
4 (2.3%)	Multiple sclerosis
3 (1.7%)	Parkinson’s disease
2 (1.1%)	Encephalopathy
2 (1.1%)	Spasticity
2 (1.1%)	Laryngeal spasm
1 (0.6%)	Status post traumatic brain injury
1 (0.6%)	Oral squamous cell carcinoma

**Table 2:** Diagnoses in subjects with psychiatric disorders (*n* = 57).

Number (Percentage)	Diagnosis
47 (82.5%)	Dental phobia not further specified
6 (10.5%)	Schizophrenia
2 (3.5%)	Personality disorder
1 (1.75%)	Attention deficit hyperactivity disorder
1 (1.75%)	Posttraumatic stress disorder

**Table 3:** Distribution (median (interquartile range)) of restored teeth, restored tooth surfaces, and extracted teeth—by gender, diagnoses, and residence.

Subgroups	Restored Teeth	Restored Tooth Surfaces	Extracted Teeth
Males	6 (2.5–9) ^a^	10 (4–15) ^b^	2 (0.5–6.5) ^c^
Females	5 (2–9) ^a^	8 (4–15) ^b^	2.5 (0–5) ^c^
Intellectual or physical disablement	5 (2–8) ^d^	8 (3–13) ^e^	1.5 (0–5) ^e^
Psychiatric disorder	7 (5–10) ^d^	14 (9–19) ^e^	5 (3–9.5) ^e^
Private home	6 (3–10) ^e^	11 (6–16) ^e^	2 (0–5) ^f^
Nursing home	3 (1–6) ^e^	7 (2–11) ^e^	4 (1–9.5) ^f^

^a^*p* = 0.55; ^b^ *p* = 0.52; ^c^ *p* = 0.58; ^d^ *p* = 0.01; ^e^ *p* < 0.001; ^f^ *p* = 0.003; Mann–Whitney *U* test.

## Data Availability

All data generated in this study is contained within this publication.
